# Non-viable *Aspergillus fumigatus* promotes chronic inflammation and angiogenesis in a murine fungus ball model

**DOI:** 10.1128/spectrum.03467-25

**Published:** 2026-03-24

**Authors:** Ryosuke Hamashima, Masato Tashiro, Yuichiro Nakano, Tomoyuki Shirahige, Hotaka Namie, Hiromu Yano, Yuya Ito, Masataka Yoshida, Tatsuro Hirayama, Kazuaki Takeda, Naoki Iwanaga, Satoshi Kakiuchi, Kodai Nishi, Hong Liu, Takahiro Takazono, Takeshi Tanaka, Akira Watanabe, Yoshihiro Komohara, Akitsugu Furumoto, Katsunori Yanagihara, Hiroshi Mukae, Scott G. Filler, Koichi Takayama, Koichi Izumikawa

**Affiliations:** 1 Department of Infectious Diseases, Nagasaki University Graduate School of Biomedical Sciences 200674 , Nagasaki, Japan; 2 Department of Pulmonary Medicine, Graduate School of Medical Science, Kyoto Prefectural University of Medicine 12898, Kyoto, Japan; 3 Infection Control and Education Center, Department of Infectious Diseases, Nagasaki University Hospital 88380, Nagasaki, Japan; 4 Department of Infectious Diseases, Yokohama City University Graduate School of Medicine 13155, Kanagawa, Japan; 5 Department of Tumor Pathology, Graduate School of Health Sciences, Kumamoto University 13205, Kumamoto, Japan; 6 Department of Respiratory Medicine, Nagasaki University Hospital 88380, Nagasaki, Japan; 7 Department of Pharmacotherapeutics, Nagasaki University Graduate School of Biomedical Sciences 200674 , Nagasaki, Japan; 8 Department of Radioisotope Medicine, Atomic Bomb Disease Institute, Nagasaki University 12961, Nagasaki, Japan; 9 Division of Infectious Diseases, Lundquist Institute for Biomedical Innovation at Harbor-UCLA Medical Center 117316 , Torrance, California, USA; 10 Infectious Diseases Treatment and Prevention Center, Department of Infectious Diseases, Nagasaki University Hospital 88380, Nagasaki, Japan; 11 Division of Clinical Research, Medical Mycology Research Center, Chiba University 12737, Chiba, Japan; 12 Department of Cell Pathology, Graduate School of Medical Sciences, Kumamoto University 13205, Kumamoto, Japan; 13 Infectious Diseases Experts Training Center, Department of Infectious Diseases, Nagasaki University Hospital 88380, Nagasaki, Japan; 14 Department of Laboratory Medicine, Nagasaki University Graduate School of Biomedical Sciences 12961, Nagasaki, Japan; 15 Department of Respiratory Medicine, Nagasaki University Graduate School of Biomedical Sciences 200674 , Nagasaki, Japan; 16 David Geffen School of Medicine at UCLA 12222 , Los Angeles, California, USA; Universidade de Sao Paulo Campus de Ribeirao Preto, Ribeirao Preto, Sao Paulo, Brazil

**Keywords:** angiogenesis, animal model, aspergilloma, *Aspergillus fumigatus*, chronic inflammation, dead hyphae, foam cell, fungus ball, lipid metabolism

## Abstract

An aspergilloma is a fungus ball that forms within a preexisting cavity, most commonly in the lungs but also in the paranasal sinuses. It represents a chronic inflammatory disorder that can persist for months and cause life-threatening hemoptysis. In this study, we hypothesized that the fungus ball is predominantly composed of non-viable hyphae and that chronic inflammation is maintained primarily by host responses to these non-viable elements rather than by ongoing fungal proliferation. To investigate this, we created a murine fungus ball model by implanting heat-killed *Aspergillus fumigatus* fungus balls, allowing for ≥16 weeks of longitudinal assessment. The model recapitulated key pathological features of the human disease, including progressive fibrosis and sustained angiogenesis, coinciding with elevated vascular endothelial growth factor (VEGF). A distinct immune response was observed where neutrophils infiltrated the fungus ball core, while macrophages accumulated at the periphery. Strikingly, these macrophages surrounding the lesion progressively became lipid-laden foam-like cells with an M1-skewed profile, a phenomenon reproduced *in vitro* by exposure to non-viable hyphae alone. Thus, the fungal material itself can sustain chronic inflammation and vascular remodeling, implicating macrophage lipid accumulation as a process potentially contributing to the chronic host response. These findings highlight lipid dysregulation and failed fungal clearance as key features of the host-response disorder associated with the fungus ball and establish a tractable platform for evaluating host-directed therapies.

**IMPORTANCE:**

Aspergilloma, a fungus ball that forms in preexisting cavities of the lung or paranasal sinuses, can cause chronic symptoms and life-threatening bleeding. Current fungicidal treatments, including antifungals, are often ineffective, and surgery to remove the fungal ball is risky. To better understand why these lesions can persist even when active fungal growth is limited, we developed a murine subcutaneous fungus ball model using non-viable *Aspergillus fumigatus*. In this model, a fungal mass composed largely of non-viable hyphae was associated with chronic inflammation, neovascularization, fibrosis, and the accumulation of lipid-laden, foam cell-like macrophages around the fungus ball. These findings support the concept that persistent non-viable fungal material can sustain key features of aspergilloma-like pathology through dysregulated host responses. They provide a conceptual rationale for future studies of host-directed strategies that modulate inflammatory and lipid-handling pathways in addition to conventional antifungal approaches.

## INTRODUCTION

Aspergilloma is characterized by the formation of a compact fungus ball by *Aspergillus* species within a preexisting cavity in the human body, most commonly in the lungs, although the paranasal sinuses and, rarely, the middle ear can also be affected (1, 2). This disease typically has a protracted course lasting over 3 months and is marked by persistent inflammation (3). Even though tissue invasion is uncommon, chronic inflammation can trigger life-threatening bleeding in up to 72% of patients (4, 5). Globally, an estimated 1.83 million individuals develop chronic pulmonary aspergillosis, which includes aspergilloma, each year (6). The 1-year mortality rate can reach 15%, and the 5-year mortality rate can be as high as 32%, underscoring the significant clinical and economic burden of this disease (7–10).

Aspergilloma can be cured by surgical resection, but this approach carries significant perioperative risks, with post-operative mortality rates of up to 5% and complications reported in up to 63% of cases (9). Moreover, many patients have compromised respiratory function due to underlying lung conditions, rendering nearly half of them ineligible for surgery (11, 12). Although antifungal therapies exist, they are often not curative and primarily serve to keep the disease under control rather than eradicate the fungus ball, highlighting the critical need for novel therapeutic strategies that circumvent the limitations of surgery (11).

Aspergilloma remains comparatively under-investigated experimentally, despite the wealth of research on invasive aspergillosis (13). Early animal models in rabbits relied on highly invasive procedures—such as bronchial and vascular ligation to induce bronchiectasis, or artificial pneumothorax to create cavities in the pleural space—and were consequently too complex for routine chronic studies (14–16). Recently, Urb et al. introduced a murine model using agar beads containing *Aspergillus fumigatus* conidia, allowing the fungus to persist for nearly 1 month in the lung (17). However, because the fungus was cleared within a month, the model fails to reproduce the chronic host–pathogen interaction lasting more than 3 months that typifies clinical aspergilloma, thereby limiting its value for studying such protracted disease processes.

We formulated two key hypotheses regarding aspergilloma pathogenesis. First, *Aspergillus* can establish a fungus ball in any organ connected to the external environment, not solely the airways—consistent with clinical observations of aspergilloma in the lungs, sinuses, and even the middle ear following tympanic membrane perforation (1, 2). Second, factors beyond organ-specific epithelium may be central to the chronic host–aspergilloma interaction. Histopathological examinations often show erosion and fibrosis of the cavity wall, coupled with loss of the original epithelial architecture (18). Thus, while the airways serve as a primary entry route for *Aspergillus* conidia, the mere presence of a preexisting air-filled cavity could be the crucial driver of fungus ball formation.

Here, we report a novel, minimally invasive murine fungus ball model that establishes a persistent fungus ball within an air-filled subcutaneous cavity for more than a month. By recapitulating the formation of an enduring fungus ball, this model enables an in-depth investigation into the long-term host–fungus interactions characteristic of aspergilloma. Our findings not only offer a simplified and reproducible platform for mechanistic studies but also pave the way for developing more effective, minimally invasive therapies.

## RESULTS

### Tissue invasion by live fungus balls occurs even in immunocompetent hosts

**Fig 1:**
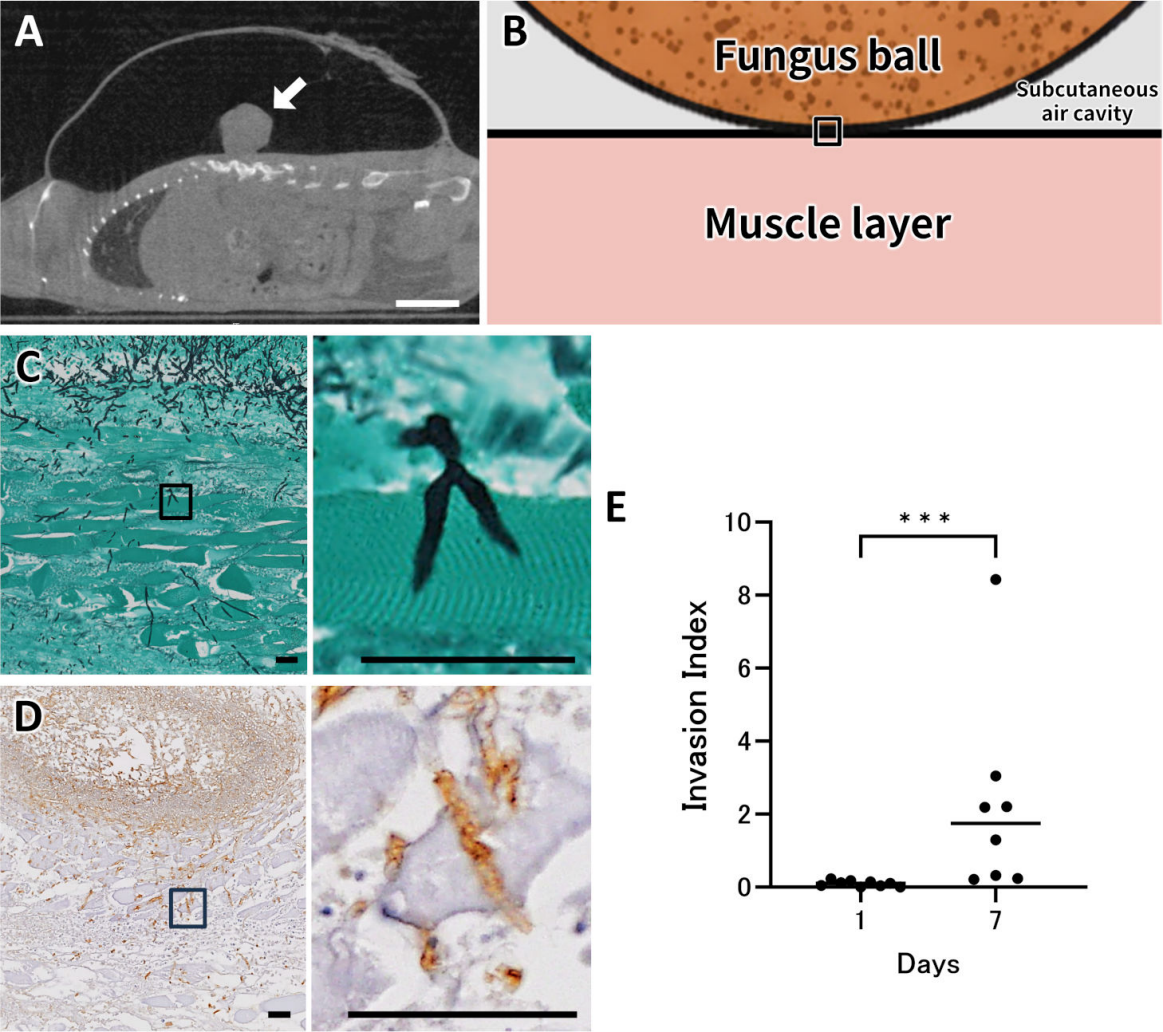
Subcutaneous implantation of a live *Aspergillus fumigatus* fungus ball in the mouse. (**A**) Sagittal computed-tomography (CT) image acquired immediately after implantation shows a fungus ball (white arrow) occupying a subcutaneous air cavity. Scale bar, 10 mm. (**B**) A schematic representation illustrating the fungus ball’s position relative to the muscle layer. The boxed area indicates the interface region shown in the subsequent pathological images in panels **C** and **D**. (**C**) GMS staining 7 days post-implantation reveals hyphal invasion (black) into the surrounding tissue; the right panel is a higher-magnification view of the boxed area. Scale bar, 40 μm. (**D**) Immunohistochemistry with an anti-*Aspergillus* antibody (brown) confirms that the invading hyphae are *Aspergillus* positive; the right panel shows a higher-magnification view of the boxed area. Scale bar, 40 μm. (**E**) Invasion index of fungal hyphae in the surrounding tissue on day 1 (*n* = 9) and day 7 (*n* = 8). Each dot represents one mouse, and horizontal bars indicate medians. Statistical significance was assessed with the Mann–Whitney U test; ***, *P* < 0.001 (day 7 vs day 1).

The initial model involved implanting live *A. fumigatus* hyphae into a subcutaneous air pocket on the dorsal surface of immunocompetent mice. The sagittal cross-sectional image of our mouse model obtained from a computed tomography scan is shown in Fig. 1A. Histopathological assessment by Grocott’s methenamine silver (GMS) staining and immunohistochemistry (IHC) for *Aspergillus* on day 7 demonstrated that fungal hyphae had begun to invade tissue, even in immunocompetent mice (Fig. 1C and D). In these mice, the majority implanted with live fungus balls eventually experienced skin or spinal cord invasion, resulting in skin lesions or spinal cord injury within 2 weeks of implantation, precluding further observations. To quantify the extent of fungal penetration, we devised an “invasion index” based on IHC (Fig. S2). By day 7, this index had increased significantly compared to day 1 (*P* < 0.001, Fig. 1E), indicating progressive tissue infiltration even during the first week post-implantation.

### Heat-killed fungus ball implantation reproduces pathological features of clinical aspergilloma

**Fig 2:**
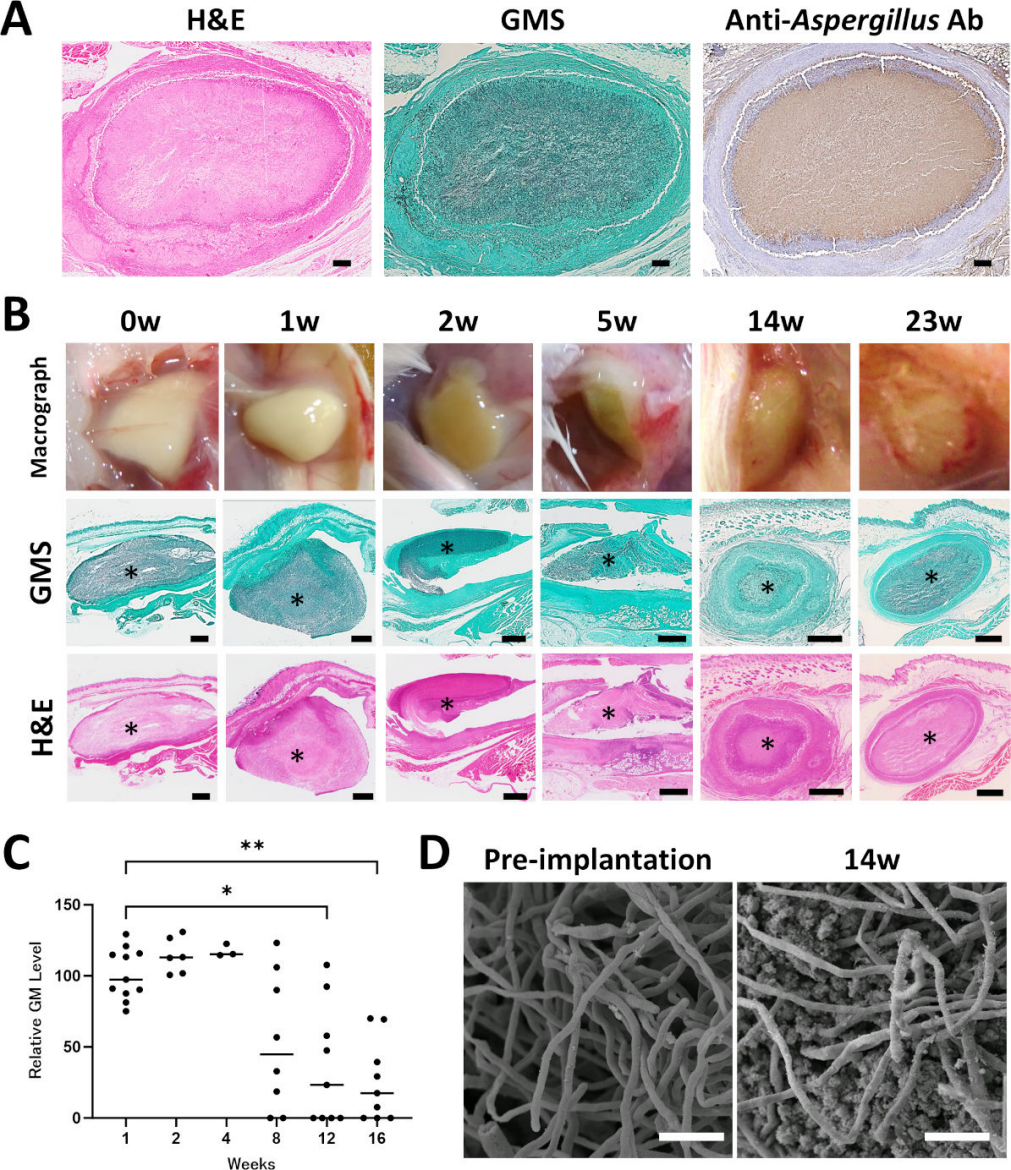
Persistence of heat-killed *Aspergillus fumigatus* fungus balls in a subcutaneous cavity. (**A**) Cross-sections of a heat-killed fungus ball, 14 weeks after implantation, stained with H&E, GMS, and an anti-*Aspergillus* antibody, demonstrate retention of the fungal mass. Scale bar, 250 μm. (**B**) Time-course images of the same model at 0 (defined as 1 day post-implantation), 1, 2, 5, 14, and 23 weeks post-implantation: macroscopic view (top), GMS stain (middle), and H&E stain (bottom); asterisks denote the fungus ball. Scale bars, 250 μm. (**C**) Relative GM level assessed by the GM index at 1, 2, 4, 8, 12, and 16 weeks (*n* = 3–11 per time point). Each dot represents one mouse; horizontal bars show medians. Statistical significance was determined with the Kruskal–Wallis test, followed by Dunn’s multiple-comparison test; *, *P* < 0.05 (week 12 vs week 1); **, *P* < 0.01 (week 16 vs week 1). (**D**) Comparison of fungus ball surface ultrastructure by scanning electron microscopy. The left panel shows the surface prior to implantation, revealing relatively clean hyphae lacking host material or extracellular matrix. The right panel shows the fungus ball surface at 14 weeks post-implantation, where hyphae appear coated or interspersed with host-derived material. Scale bars, 10 μm. Images at 0, 2, and 14 weeks in panel B (GMS) are reproduced in Fig. 5C solely to indicate the locations of the corresponding higher-magnification views.

To avoid tissue invasion, heat-killed fungus balls were implanted into immunocompetent mice. This approach allowed long-term observation without the severe morbidity, such as spinal cord injury, seen in the live fungus model. At week 14, histopathological analysis demonstrated persistent dead fungus balls within a subcutaneous cavity, exhibiting pathological features that closely resembled those observed in human clinical aspergilloma samples (Fig. 2A). Over the course of observation, the heat-killed fungus balls initially appeared white up to the first week. By the second week, they had turned yellow-brown with purulent characteristics (Fig. 2B). From weeks 14 to 23, hematoxylin and eosin (H&E) showed the fungus balls were gradually encapsulated by a membrane-like structure (Fig. 2B). To quantitatively assess changes in the relative galactomannan level over time, galactomannan (GM) concentrations in the supernatants of homogenized fungus ball suspensions were measured. Consistent with the histopathological findings, GM was detectable within the fungus balls for over 3 months, although the overall relative galactomannan level gradually decreased (Fig. 2C). The median relative galactomannan level decreased between week 1 and week 16, corresponding to 82% reduction (*P* < 0.01, Fig. 2C), but the fungus ball itself was still macroscopically present in 67% of mice at week 16. Scanning electron microscopy (SEM) analysis revealed exposed hyphal structures essentially free of extracellular matrix (ECM) or other surface components on the heat-inactivated fungus balls prior to implantation (Fig. 2D, left panel). In contrast, by 14 weeks post-implantation, the fungus ball surface showed hyphae coated or interspersed with host-derived material (Fig. 2D, right panel).

### Neovascularization and fibrotic remodeling develop around the fungus ball

**Fig 3:**
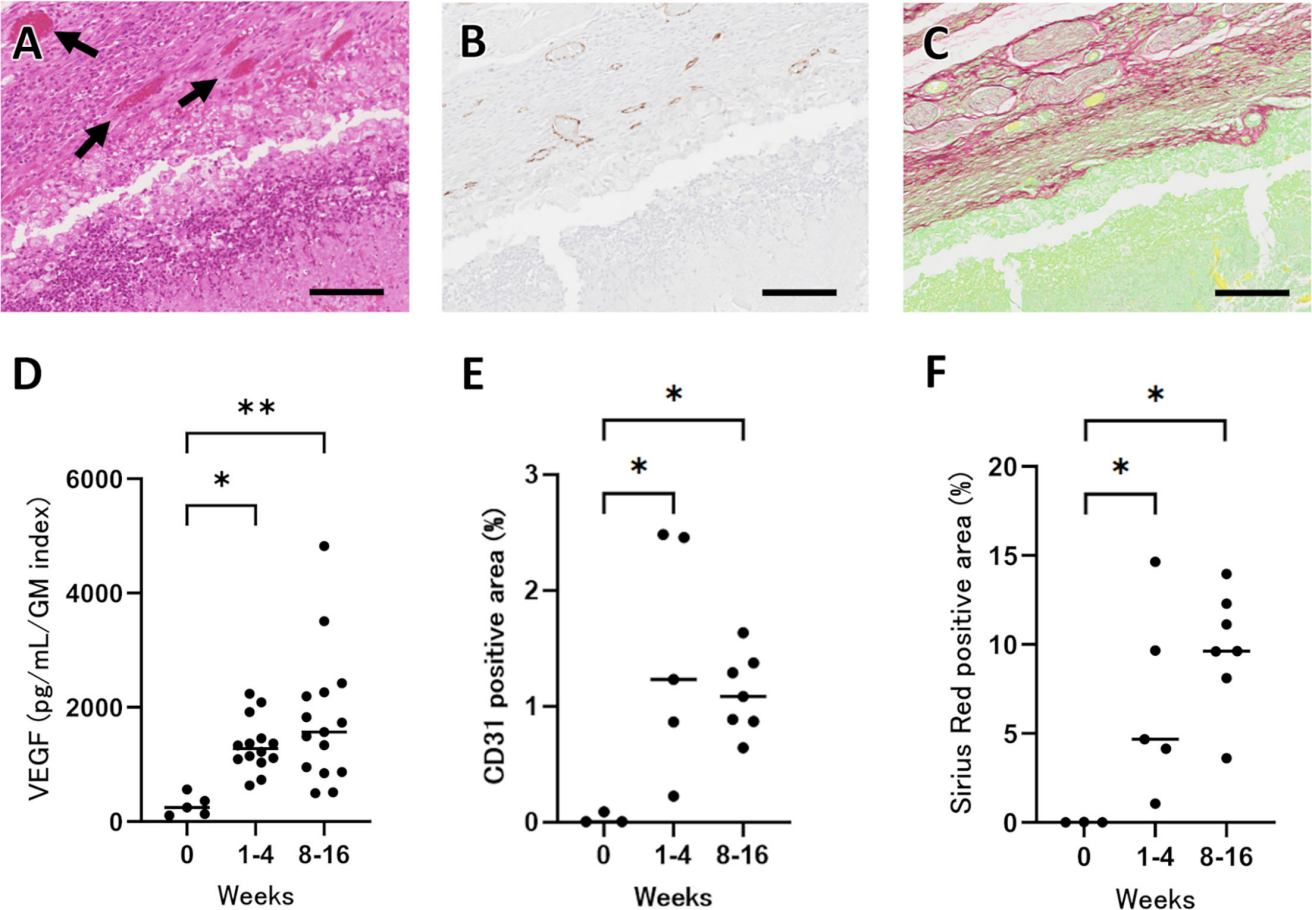
Tissue remodeling surrounding a heat-killed *Aspergillus fumigatus* fungus ball. (**A**) H&E section 14 weeks after implantation shows fibrovascular tissue (arrows) abutting the fungus ball. (**B**) CD31 immunohistochemistry (brown) in the same region demonstrates neovascularization. (**C**) Sirius Red staining reveals collagen deposition (red) consistent with fibrosis. Scale bars, 100 μm. (**D**) VEGF levels, normalized to GM index, at week 0 (*n* = 5), weeks 1–4 (*n* = 14), and weeks 8–16 (*n* = 15). (**E**) CD31-positive area (%) and (**F**) Sirius Red-positive area (%) at the same time points: week 0 (*n* = 3), weeks 1–4 (*n* = 5), and weeks 8–16 (*n* = 7). Each dot represents one mouse; horizontal bars indicate medians. Statistical significance was assessed with the Kruskal–Wallis test, followed by Dunn’s multiple-comparison test; *, *P* < 0.05; **, *P* < 0.01 vs week 0.

Histopathological examination revealed marked tissue remodeling surrounding the heat-killed fungus balls. Fibrovascular tissue was observed at the interface between the fungus ball and host tissue (Fig. 3A, arrow), and immunostaining for the endothelial cell marker CD31 confirmed the formation of new vascular networks in the surrounding tissue (Fig. 3B). In addition, Sirius Red staining demonstrated abundant collagen deposition around the fungus balls (Fig. 3C). To quantify these changes, the concentration of vascular endothelial growth factor (VEGF), normalized to the GM index, was measured at various time points. VEGF levels, relative to the remaining fungal mass, significantly increased from baseline through weeks 1–4 (*P* < 0.05 vs week 0, Fig. 3D), and further increased during weeks 8–16 (*P* < 0.01 vs week 0, Fig. 3D). Similarly, the area of CD31-positive staining increased markedly from weeks 1–4 (*P* < 0.05 vs week 0, Fig. 3E) and remained elevated through weeks 8–16 (*P* < 0.05 vs week 0, Fig. 3E). The Sirius Red-positive area also increased over time, suggesting progressive collagen deposition around the fungus balls (Fig. 3F).

### Neutrophils infiltrate the interior of the fungus ball over time

**Fig 4:**
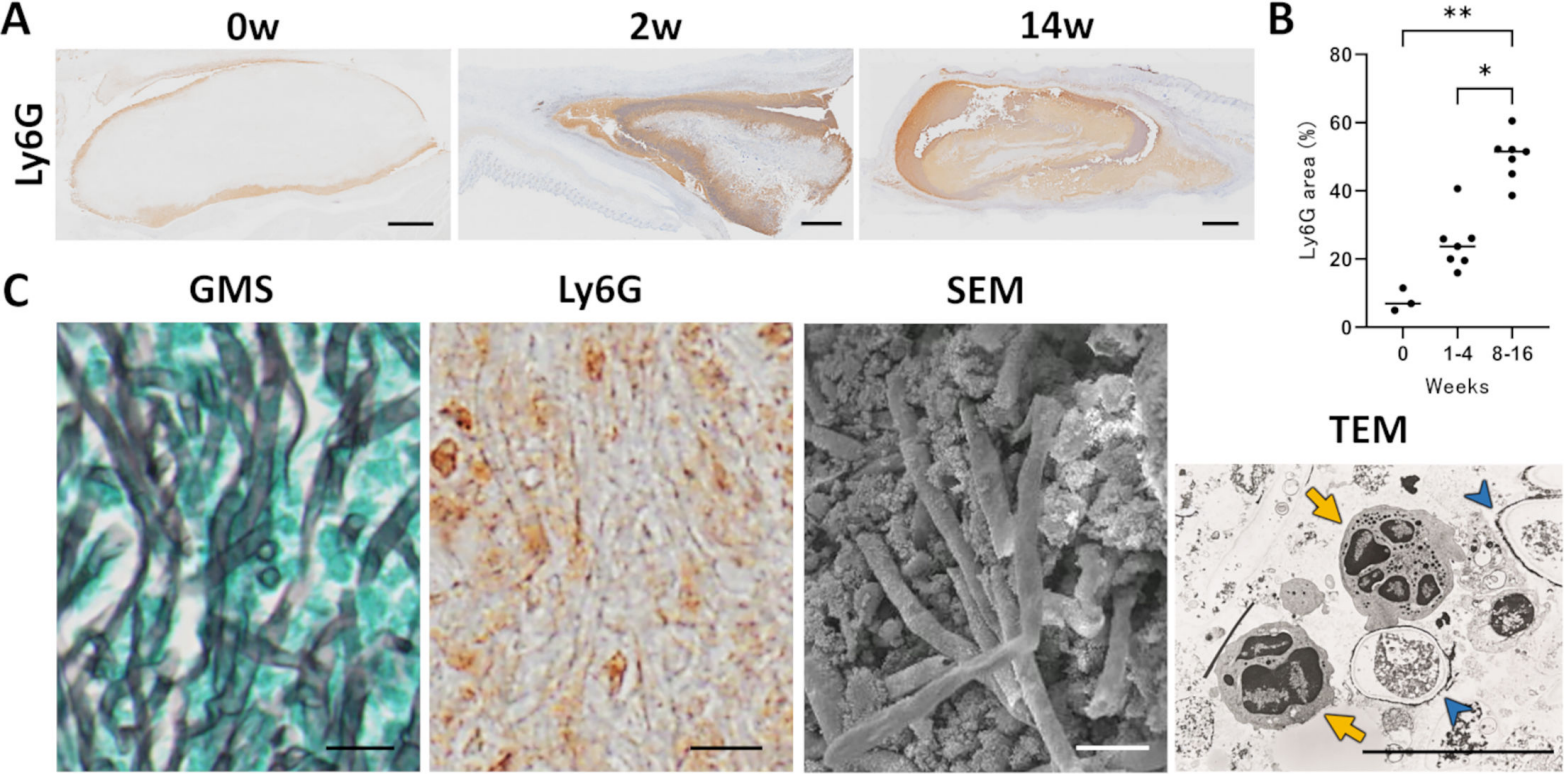
Neutrophil infiltration into a heat-killed *Aspergillus fumigatus* fungus ball. (**A**) Ly6G immunohistochemistry (brown) at 0, 2, and 14 weeks post-implantation demonstrates progressive neutrophil accumulation within the fungus ball. Scale bars, 1 mm. (**B**) Ly6G-positive area (%) at week 0 (*n* = 3), weeks 1–4 (*n* = 7), and weeks 8–16 (*n* = 7). Each dot represents one mouse; horizontal bars indicate medians. Statistical significance was assessed with the Kruskal–Wallis test, followed by Dunn’s multiple-comparison test; *, *P* < 0.05 (weeks 8–16 vs weeks 1–4); **, *P* < 0.01 (weeks 8–16 vs week 0). (**C**) Detailed correlative analysis of the fungus ball interior (at 14 weeks post-implantation). From left to right: GMS staining highlights fungal hyphae (black). Ly6G immunohistochemistry (brown) confirms that neutrophils surround the hyphae. SEM reveals dense networks of hyphae interspersed with granular aggregates corresponding to neutrophils. TEM shows host neutrophils (yellow arrows) adhering to *A. fumigatus* hyphae (blue arrowheads). Scale bars, 10 μm.

Neutrophils were recruited immediately after implantation and progressively infiltrated into the fungus ball over time, as illustrated by Ly6G IHC (Fig. 4A and B). A detailed correlative analysis of the fungus ball interior at 14 weeks confirmed the close association between hyphae and neutrophils (Fig. 4C). GMS staining highlighted the fungal hyphae, while corresponding Ly6G immunohistochemistry confirmed that neutrophils surrounded these hyphae. SEM revealed the ultrastructure of dense hyphal networks interspersed with these granular neutrophil aggregates. Transmission electron microscopy (TEM) further visualized neutrophils (yellow arrows) in direct contact with *A. fumigatus* hyphae (blue arrowheads) (Fig. 4C).

### Macrophages accumulate on the fungus ball surface and phagocytose fungal body

**Fig 5:**
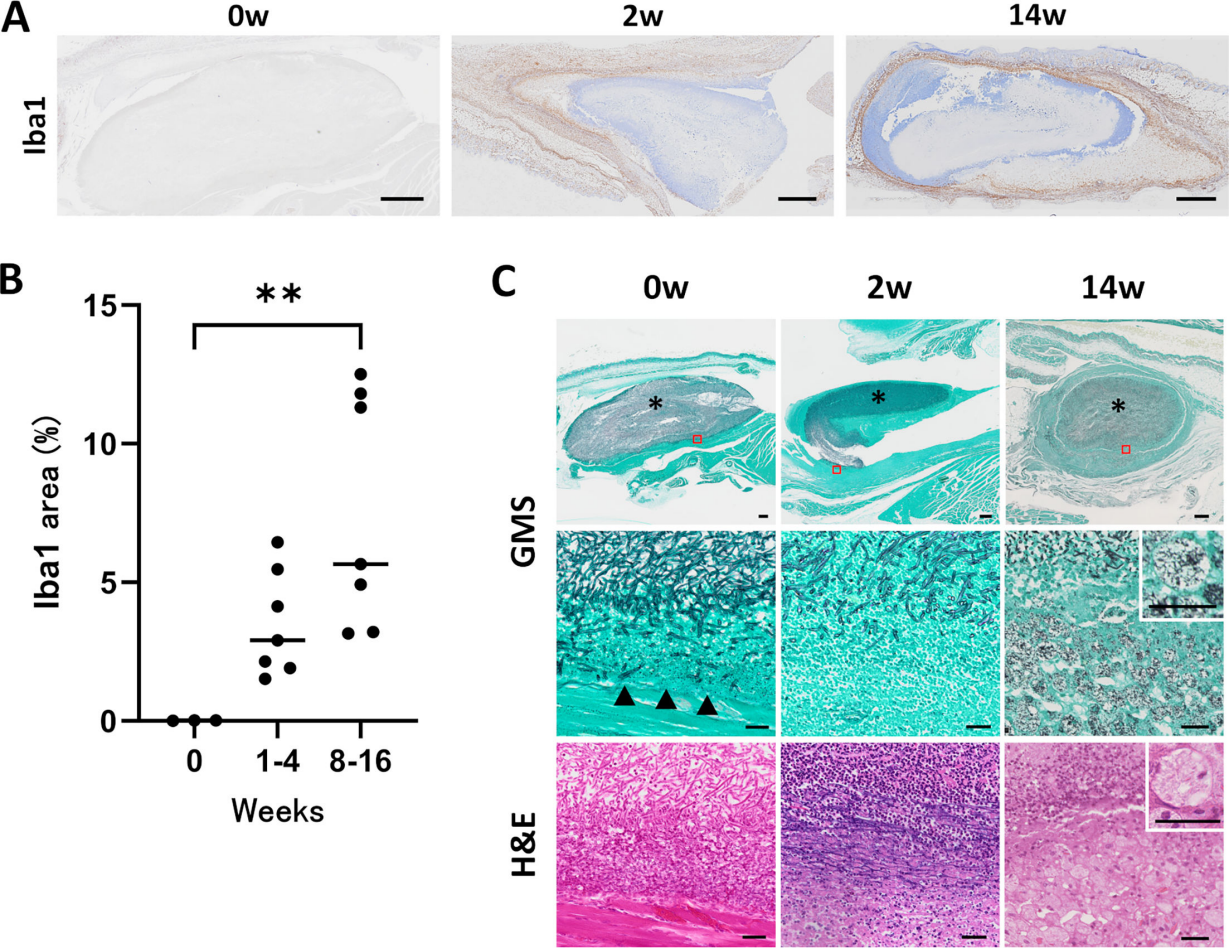
Macrophage accumulation on the surface of a heat-killed *Aspergillus fumigatus* fungus ball. (**A**) Iba1 immunohistochemistry (brown) at 0, 2, and 14 weeks post-implantation demonstrates progressive recruitment of macrophages to the periphery of the fungus ball. Scale bars, 1 mm. (**B**) Iba1-positive area (%) in the fungus ball wall at week 0 (*n* = 3), weeks 1–4 (*n* = 7), and weeks 8–16 (*n* = 7). Each dot represents one mouse; horizontal bars denote medians. Statistical significance was determined with the Kruskal–Wallis test, followed by Dunn’s multiple-comparison test; **, *P* < 0.01 (weeks 8–16 vs week 0). (**C**) GMS (top) and H&E (bottom) sections at 0, 2, and 14 weeks. Asterisks mark the fungus ball. Red boxes indicate regions shown at higher magnification (middle panels), where fragmented hyphae (black arrowheads, week 0) are progressively phagocytosed by infiltrating macrophages that transform into foam-like cells (insets, week 14). Scale bars, 40 μm. In panel C, the low-magnification GMS images at 0, 2, and 14 weeks are reproduced from Fig. 2B for orientation; they are shown again only to indicate the regions used for the higher-magnification views.

In contrast to neutrophils, macrophage infiltration was prominent, particularly at the peripheral interface of the fungus ball. IHC for the macrophage marker Iba1 revealed little to no signal at week 0, but robust staining became evident by 2 weeks and persisted through 14 weeks (Fig. 5A). Quantitative image analysis indicated that Iba1-positive area increased significantly by weeks 8–16 (*P* < 0.01 vs week 0, Fig. 5B). Closer histological inspection (GMS and H&E) confirmed that, over time, macrophages progressively colonized the surface layers of the fungus ball (Fig. 5C, top row). Early after implantation (0–2 weeks), multiple fragmented fungal hyphae were observed near the surface (Fig. 5C, arrowheads). By week 14, macrophages had phagocytosed these hyphae and their fragments, leading to intracellular debris accumulation and a characteristic foam-like morphology (Fig. 5C, middle and bottom insets).

### Macrophages harbor lipid droplets and exhibit a foam cell-like immunophenotype

**Fig 6:**
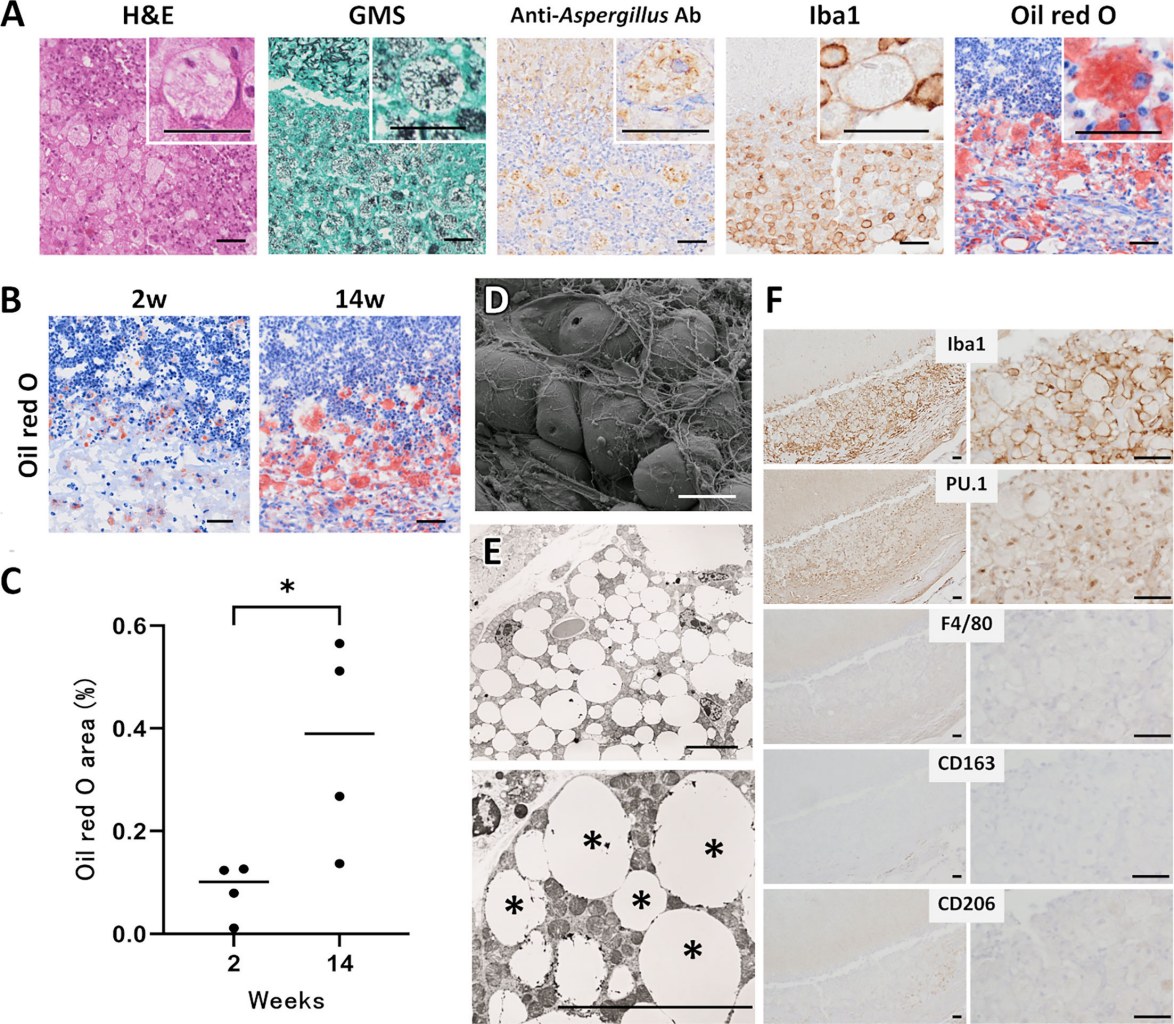
Foamy macrophages at the surface of a heat-killed *Aspergillus fumigatus* fungus ball. (**A**) Correlative staining of the lesion periphery at 14 weeks post-implantation. The H&E and GMS micrographs are the same as the week 14 higher-magnification images shown in Fig. 5C and are reproduced here to enable side-by-side comparison. Anti-Aspergillus antibody and Iba1 panels are from serial paraffin sections of the same lesion region. The Oil Red O image is from a frozen section prepared from the same time point, shown to illustrate lipid accumulation in foam-like macrophages. Insets show higher-magnification views. Scale bars, 40 μm. (**B**) Oil Red O staining at 2 and 14 weeks demonstrates progressive lipid accumulation in surface macrophages (red); insets highlight foam-like cells with prominent lipid droplets. Scale bar, 40 μm. (**C**) Oil Red O-positive area (%) at 2 weeks (*n* = 4) and 14 weeks (*n* = 4). Each dot represents one mouse; horizontal bars indicate medians. Statistical significance was assessed with the Mann–Whitney U test; *, P < 0.05 (week 14 vs week 2). (**D**) Scanning electron micrograph of the fungus ball surface reveals numerous balloon-shaped macrophage-like cells entangled in fibrous material. Scale bar, 40 μm. (**E**) Transmission-electron micrograph confirms enlarged macrophages containing abundant intracellular lipid droplets (*). Scale bar, 10 μm. (**F**) Consecutive immunohistochemical sections at 14 weeks show that surface macrophages are positive for Iba1 and PU.1 but negative for F4/80, CD163, and CD206, consistent with a foam-like phenotype. Scale bars, 40 μm.

To first confirm that these macrophages were engaging with fungal elements, we stained tissue sections with an anti-*Aspergillus* antibody. At 14 weeks, this revealed numerous fungal remnants within the cytoplasm of macrophages at the lesion periphery (Fig. 6A). Given the vacuolated cytoplasm of the macrophages at the peripheral interface of the fungus ball, we used Oil Red O staining to detect lipid droplets. By 2 weeks post-implantation, surface macrophages showed a few lipid inclusions (Fig. 6B). By 14 weeks, many had developed abundant intracellular lipid bodies visible in light microscopy (Fig. 6B, insets), while SEM revealed their corresponding swollen, balloon-like morphology (Fig. 6D). Quantification revealed a significant rise in Oil Red O-positive area between 2 and 14 weeks (*P* < 0.05, Fig. 6C). TEM demonstrated that these foam cell-like macrophages harbored numerous lipid vacuoles interspersed (Fig. 6E). Furthermore, consecutive sections stained for Iba1, PU.1, F4/80, CD163, and CD206 showed that these foam cell-like macrophages robustly expressed Iba1 and PU.1 but were largely negative for F4/80, CD163, and CD206, suggesting a distinct activation state associated with a foam-like macrophage phenotype (Fig. 6F).

### Heat-killed *A. fumigatus* hyphae induce macrophage cytotoxicity and foam cell formation *in vitro*

**Fig 7:**
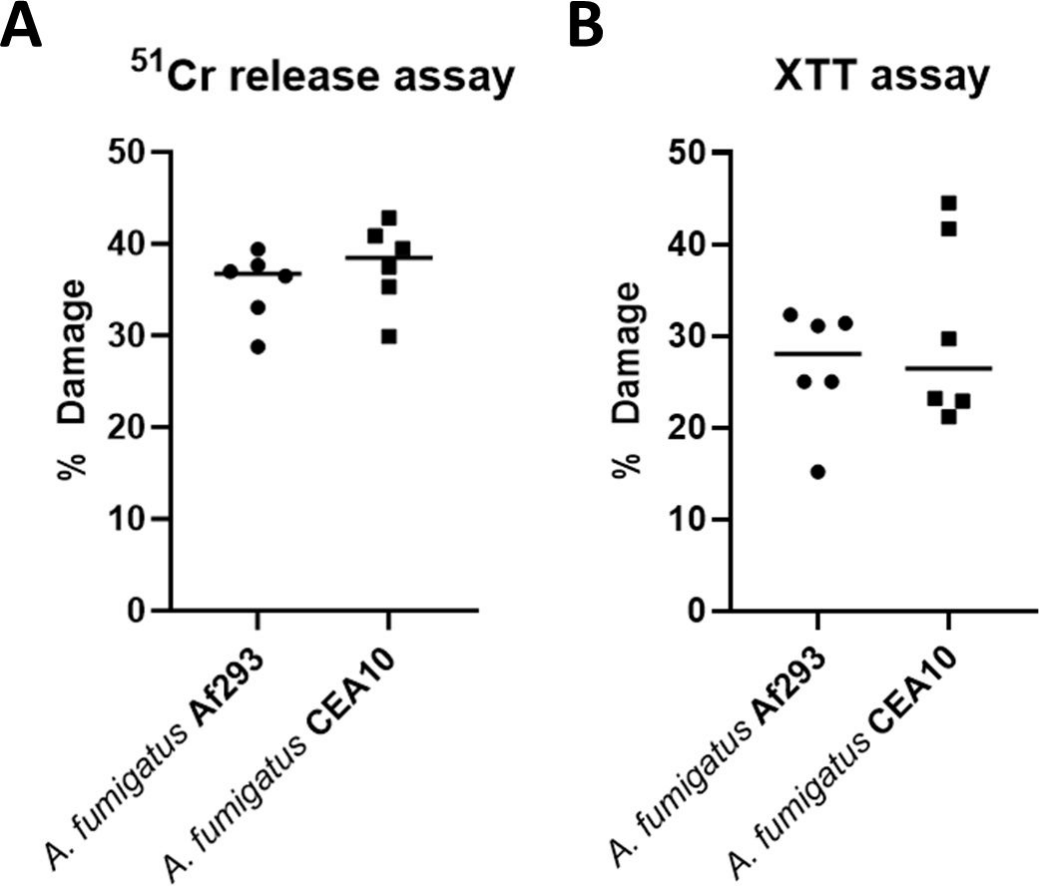
Cytotoxicity of heat-killed *Aspergillus fumigatus* hyphae toward RAW264.7 macrophages. RAW264.7 cells were incubated for 24 h with heat-killed hyphae from strain Af293 or CEA10. Cytotoxicity was quantified by ^51^Cr release (**A**) and XTT assay (**B**). Each symbol represents one independent experiment (*n* = 6); horizontal bars denote medians. No significant difference was detected between the two strains (Mann–Whitney U test).

**Fig 8:**
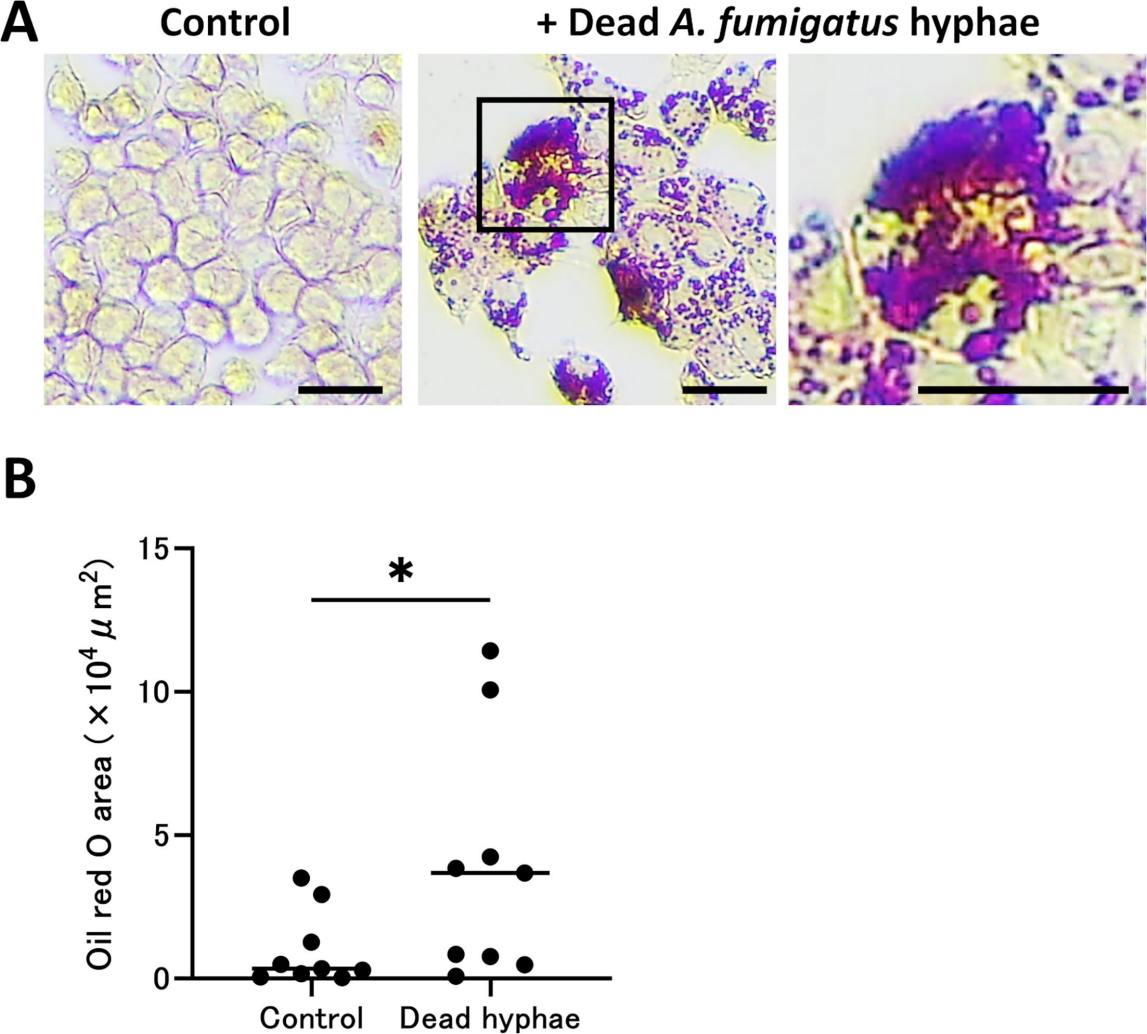
Heat-killed *Aspergillus fumigatus* hyphae induce foam-cell formation in RAW264.7 macrophages. (**A**) RAW264.7 cells were incubated for 24 h with or without heat-killed *A. fumigatus* hyphae and stained with Oil Red O (red) to visualize intracellular lipid droplets. Control cells show minimal staining, whereas hyphae-exposed cells contain abundant lipid droplets; the right panel is a higher-magnification view of the boxed region. Scale bars, 20 μm. (**B**) Quantification of Oil Red O-positive area (×10^4^ µm²) in control and dead hyphae-exposed cultures (*n* = 9 independent experiments). Each dot represents an individual experiment, and horizontal bars indicate the median. Statistical analysis was performed with the Mann–Whitney U test; *, *P* < 0.05 (dead hyphae vs control).

To further assess the host–fungus interactions underlying foam cell development, we exposed the murine macrophage line RAW264.7 to heat-killed *A. fumigatus* hyphae *in vitro*. A ^51^Cr release assay and XTT assay demonstrated that heat-killed hyphae from two *A. fumigatus* strains (Af293 and CEA10) induced similar levels of macrophage damage (Fig. 7A and B). Thus, even non-viable fungal structures can elicit cytotoxic effects on host macrophages. In addition, co-culturing RAW264.7 cells with heat-killed hyphae for 24 h also triggered robust foam cell formation, as evidenced by abundant Oil Red O-stained lipid droplets (Fig. 8A). Quantification of lipid area revealed a significant increase compared to control cultures (*P* < 0.05, Fig. 8B).

## DISCUSSION

This model recapitulates hallmark features of human aspergilloma, including a neutrophil-rich core and surrounding fibro-angiogenic rim, which show histopathological similarity to human clinical samples (18, 19). Crucially, the model also revealed a previously unrecognized feature—lipid-laden foam cell macrophages—highlighting a potential role for dysregulated lipid metabolism in this chronic disease pathology.

In our model, live fungus balls rapidly breached the cavity wall, often causing skin or spinal cord damage within 14 days, precluding chronic phase analysis. In contrast, implantation of heat-killed fungus balls enabled long-term observation without tissue invasion. These findings are consistent with clinical observations showing that fungal cultures of surgically resected aspergillomas are positive in only about 20% of cases and that histology often demonstrates poorly staining hyphae within the lesion, indicating that most aspergillomas contain largely non-viable fungus (19, 20). Unlike prior murine models that relied on viable hyphae but failed to induce chronic disease, our system maintained a stable lesion for over 3 months. This persistence provides a robust platform for investigating the chronic pathology of aspergilloma and testing therapeutic strategies.

A prominent feature of our model is the development of a dense fibrovascular rim surrounding the fungus ball, closely resembling the cavity wall in human aspergilloma (18). Sirius Red staining showed progressive collagen deposition, while CD31 immunohistochemistry and VEGF quantification indicated sustained angiogenesis. Interestingly, VEGF remained elevated despite fungal non-viability, suggesting that even a diminishing amount of persistent dead fungal elements may be sufficient to maintain angiogenic signaling in the surrounding tissue. Clinically, excessive VEGF expression is associated with hemoptysis in aspergilloma patients (21).

In this model, a biphasic innate immune response was observed, wherein neutrophils infiltrated the core of the fungus ball, while macrophages accumulated at the periphery. This distribution of inflammatory cells is similar to the histopathology of human sinus fungus balls, which have also been reported to be dominated by neutrophils and macrophages (22). These findings suggest that the incompletely cleared, non-viable fungal mass acts as a chronic stimulus, eliciting a prolonged host immune response characterized by the persistent accumulation and activation of neutrophils and macrophages. However, the functional analysis of these cells in our study was limited to histological observation, and a more detailed evaluation of their functions, such as phagocytic capacity and the production of inflammatory mediators, awaits further investigation.

A central finding of this study is the progressive transformation of peripheral macrophages into lipid-laden foam cells. These cells accumulated undigested fungal debris and lipids, suggesting that even the killed fungi may compromise effective clearance mechanisms of macrophages. Similar macrophages have been documented in fine-needle biopsy specimens from human fungal infections, in which one study reported that foam cells were observed in 75% of 26 cases (16 of which were aspergillosis) (23). Additionally, *in vitro* work has shown that heat-killed *Candida albicans* can drive macrophage foam cell formation (24). We also confirmed that even non-viable *A. fumigatus* hyphae can induce macrophage damage and robust lipid accumulation. To our knowledge, this is the first murine model to demonstrate this phenomenon *in vivo* in a sustained, non-invasive context. These findings support a model in which persistent non-viable fungal elements are associated with impaired macrophage function and disrupted lipid homeostasis. This defective lipid handling may involve impaired lipid efflux, lysosomal overload, or upregulation of lipid-binding proteins, such as FABP4, as previously implicated in foam cell formation in fungal infections (24, 25). However, further profiling is needed to confirm the functional polarization status of these foam-like cells.

Paradoxically, chronic fibrotic lesions are often dominated by anti-inflammatory M2 macrophages (26). Yet, in our model, despite the presence of a prominent fibrovascular rim encasing the fungus ball, the lipid-laden macrophages within this zone displayed an M1-like CD163⁻/CD206⁻ phenotype, similar to foam cells seen in atherosclerosis (27). We acknowledge, however, that this classification is based on a limited set of surface markers (F4/80, CD163, and CD206) and that a more definitive phenotyping would require analysis of key transcription factors or cytokine gene expression. Consistent with this, *in vitro* work has shown that β-glucan engagement of Dectin-1 can drive immunosuppressive macrophages to convert to an M1-like state (28). We hypothesize that persistent exposure to fungal components, such as β-glucan or ergosterol, even in heat-killed hyphae, repolarizes macrophages toward a sustained M1-like phenotype, impairing debris clearance and perpetuating VEGF-driven angiogenesis. This feedback loop may underlie the chronicity and vascular proliferation seen in aspergilloma lesions. Future *in vitro* studies are planned to test this hypothesis directly, for example, by using Dectin-1 or Dectin-2 deficient macrophages or by stimulating cells with specific PAMPs (e.g., β-glucan, galactomannan) to assess their specific roles in lipid accumulation and polarization (29). Further investigation into the role of key transcription factors like PU.1, which was shown to be positive in the infiltrating macrophages, in mediating Dectin-1-driven responses and macrophage polarization will be crucial.

Our model has several limitations. First, our subcutaneous model in immunocompetent mice does not fully replicate the complex pulmonary environment (e.g., airflow, surfactant) or the underlying comorbidities (e.g., prior tuberculosis) often present in patients with chronic pulmonary aspergillosis. Additionally, the implanted ball becomes fixed to the cavity wall, whereas clinical aspergillomas often remain mobile within cavitary lesions, potentially altering the duration and intensity of host–fungus contact (30). Furthermore, the composition of the implanted fungus ball presents several caveats. Because the fungus ball is pre-assembled *in vitro* before implantation, the model bypasses the early establishment phases of infection. It also lacks the fungal-derived ECM that typifies clinical biofilms (31, 32). Crucially, the model presented here uses non-viable hyphae. While this allows for the study of host response to persistent fungal material, we are developing a hybrid model—seeding live *A. fumigatus* onto the dead ball core—to investigate factors related to viable fungus in a controlled, chronic setting. Methodologically, this study lacks a non-fungal control, such as inert agar beads, to distinguish fungal-specific responses from a general foreign body reaction. We also utilized a single clinical isolate (MF367) *in vivo*, and future studies using common laboratory strains are needed to confirm the generalizability of these findings.

In summary, we established a reproducible murine model that captures key histopathological features of chronic aspergilloma, including a neutrophil-rich core, a fibro-angiogenic rim, and lipid-laden foam cell-like macrophages. Our model revealed that persistent non-viable fungal debris is associated with chronic inflammation, angiogenesis, fibrosis, and foam cell formation in a murine subcutaneous cavity. Together with the absence of viable hyphae, these findings support the concept that long-lived fungal material can sustain many features of aspergilloma-like pathology, although contributions from non-specific foreign body responses cannot be excluded. These observations support the view that immune dysregulation and failed clearance of largely non-viable fungal material are important components of aspergilloma pathology, complementing the traditional focus on ongoing fungal growth. Our model provides a valuable platform for exploring macrophage lipid-handling pathways and testing adjunctive therapies aimed at accelerating cavity sterilization and preventing hemoptysis.

## MATERIALS AND METHODS

### Mice

Female ICR mice aged 6 to 9 weeks were purchased from Japan SLC, Inc. (Shizuoka, Japan). The animals were maintained under standardized, sterile environmental conditions (room temperature, 24°C; relative humidity, 50%; 12 h light-dark cycle) with free access to food and water.

### Preparation of fungus balls

*A. fumigatus* MF367, a clinical isolate from a patient with chronic pulmonary aspergillosis (33), was cultured on potato dextrose agar at 30°C for 3 to 7 days. Conidia were collected by rinsing the culture plates with phosphate-buffered saline (PBS) containing 1% Tween 20 (FUJIFILM Wako Pure Chemical, Tokyo, Japan). The conidial suspension was adjusted to 1 × 10^5^ cells/mL, and 200 µL of this suspension was added to 20 mL of potato dextrose broth (PDB) and incubated on a rotary shaker at 250 rpm for 48 h at 30°C.

Fungus balls ranging from 3 to 7 mm in diameter formed spontaneously. Those approximately 5 mm in diameter were selected using a sterile loop with an inner diameter of 5 mm to minimize size variation, transferred to 20 mL of fresh PDB, and further incubated for 24 h at 30°C to obtain fungus balls (approximately 10 mm in diameter). If heat-killed fungus balls were needed, they were autoclaved at 121°C for 15 min. Loss of viability was confirmed by the absence of fungal growth in culture. Prior to implantation, fungus balls were transferred to a tube containing 0.9% saline and washed thoroughly using a vortex mixer. This washing step was repeated twice.

### Fungus ball implantation into mice 

On day 0, mice were anesthetized by intraperitoneal injection of medetomidine (Meiji Seika Pharma Co. Ltd., Tokyo, Japan; 0.75 mg/kg), midazolam (Sandoz, Holzkirchen, Germany; 4.0 mg/kg), and butorphanol (Meiji Seika Pharma Co. Ltd., Tokyo, Japan; 5.0 mg/kg). The dorsal skin (~1 cm lateral to the midline) was sterilized with 70% ethanol, and a small incision (~5 mm) was made, and a subcutaneous pocket was bluntly dissected. A single fungus ball was gently placed into the cavity using sterile forceps, and the incision was closed with 3–4 sutures. To keep the pocket inflated, approximately 5 mL of room air was reinjected into the cavity two to three times per week thereafter; mice were lightly anesthetized with isoflurane during this reinjection procedure to minimize distress. The procedure did not affect feeding, grooming, or spontaneous activity. Post-operative analgesia (buprenorphine, 0.05 mg/kg, administered subcutaneously post-surgery and repeated if signs of pain, such as hunched posture or reduced activity, were observed) was provided.

### Histopathological and immunopathological staining

Mice were euthanized for tissue collection. Due to the difficulty of handling mice with a dorsal air cavity, animals were first induced with isoflurane, followed by an intraperitoneal injection of a three-drug anesthetic cocktail (medetomidine 0.75 mg/kg, midazolam 4.0 mg/kg, and butorphanol 5.0 mg/kg) at a volume of 400 μL per mouse. Immediately thereafter, mice received a lethal intraperitoneal injection of secobarbital sodium (approximately 150 mg/kg), referencing the reported LD50 (34). The fungus balls, together with surrounding tissues, were dissected. The tissues were fixed with 10% formalin, embedded in paraffin, and sectioned at 3 µm (the fungus ball was sliced at its largest cross-section). Sections were stained with GMS and H&E. Additional IHC was performed as previously described (18).

For IHC, the following primary antibodies were used to detect specific cell types and properties: anti-Ly6G (rabbit monoclonal; Abcam, Cambridge, UK; catalog no. ab238132) to detect neutrophils; anti-Iba1 (rabbit polyclonal; FUJIFILM Wako, Tokyo, Japan; catalog no. 019-19741), anti-PU.1 (rabbit polyclonal; Abcam, Cambridge, UK; catalog no. ab230336), anti-F4/80 (rat monoclonal; Bio-Rad, Hercules, CA, USA; clone CI:A3-1), anti-CD163 (rabbit monoclonal; Abcam, Cambridge, UK; clone EPR19518), and anti-CD206 (rabbit polyclonal; Abcam, Cambridge, UK; catalog no. ab64693) to detect macrophage properties; anti-CD31 (rabbit monoclonal; Abcam, Cambridge, UK; catalog no. ab182981) to detect vascular endothelial cells; and anti-*Aspergillus* (rabbit polyclonal; Bio-Rad, Hercules, CA, USA; catalog no. 0771-1300) to visualize *Aspergillus* hyphae. Primary antibody binding was detected by horseradish peroxidase-labeled secondary antibodies (Nichirei Biosciences, Tokyo, Japan) and visualized using 3,3′-diaminobenzidine (DAB) substrate (Nichirei Biosciences).

To evaluate lipid accumulation, mice were euthanized, and the fungus balls and adjacent tissues were removed and fixed in 4% paraformaldehyde, cryoprotected in 30% sucrose, and embedded in OCT compound to be prepared as frozen sections. Lipids were visualized by Oil Red O staining.

After rinsing with distilled water, the sections were immersed in 60% isopropanol for 20–30 s. Next, the sections were stained using the Oil Red O stain kit (Bio Mirai Koubou, Tokyo, Japan) for 60 min. They were then washed in 60% isopropanol, followed by distilled water. Afterward, the sections were stained with Mayer’s hematoxylin, rinsed in water for 10 min, and coverslipped.

Additionally, to assess fibrotic areas around the fungus balls, sections were stained with Sirius Red. Briefly, paraffin-embedded sections were deparaffinized, rehydrated, and incubated with a Sirius Red solution, followed by washing and counterstaining with hematoxylin. This staining allowed for the visualization and evaluation of collagen deposition in the fibrotic regions surrounding the fungus balls.

### Quantitative image analysis of host immune response

Slides (GMS, H&E, Oil Red O, Sirius Red, and IHC) were scanned using a slide scanner (VS200, Olympus) and viewed with OlyVIA software (Olympus Olyvia 3.4). Fiji (ImageJ version 2.9) was used to quantify neutrophils, macrophages, lipid droplet-containing cells, and fibrotic areas in and around the fungus ball. Quantification of Ly6G-positive area (Fig. 4B), Iba1-positive area (Fig. 5B), Oil Red O-positive area (Fig. 6C), and Sirius Red-positive area (Fig. 3F) was performed using Fiji (ImageJ v2.9) (35).

Briefly, a 500 µm margin from the outer edge of the fungus ball was defined using a freehand selection tool (Fig. S1A). Outside regions were cleared (Clear Outside command) (Fig. S1B), and the Threshold Color plugin was used to isolate positive DAB, Oil Red O, or Sirius Red staining (Fig. S1C). A threshold was selected to demarcate positive immunostaining. A separate threshold defined the total fungus ball plus margin area (Fig. S1D). The percentage positive area was calculated as (Positive-stained area/Total selected area) × 100.

### Quantitative image analysis of *Aspergillus* invasion

This analysis was performed only on tissues from mice implanted with live fungus balls, as infiltration does not occur in the heat-killed fungus ball group. Formalin-fixed, paraffin-embedded tissue sections were subjected to IHC staining to detect *A. fumigatus* using an anti-*Aspergillus* antibody. Tissue regions displaying continuous contact of ≥500 µm in length between the IHC-positive fungus ball and surrounding host tissue were identified. From the interface between the fungal mass and the tissue, a virtual boundary was defined 100 μm toward the tissue side. Next, five random square regions of interest (ROI), each measuring 500 μm on a side (500 × 500 µm), were selected along this boundary. The area of IHC-positive staining within each ROI was quantified using Fiji (ImageJ version 2.9). To derive the Invasion Index, the measured IHC-positive area in each ROI was divided by 500, and the mean value from the five ROIs was reported as the final index (Fig. S2).

### Electron microscopy

Fungus balls were analyzed by SEM and TEM to observe their ultrastructural characteristics. For SEM, the fungus balls were fixed in 2.5% glutaraldehyde (TAAB Laboratories Equipment Ltd., Aldermaston, UK) in PBS at 4°C for 24 h, rinsed in PBS, and then postfixed in 1% osmium tetroxide (Heraeus Chemicals, South Africa) for 2 h. After dehydration in a graded ethanol series and critical point drying, the samples were mounted on metal stubs and sputter-coated with a thin layer of gold. SEM images were obtained using a JSM-6700F scanning electron microscope (JEOL Ltd., Tokyo, Japan) at an accelerating voltage of 5–15 kV. For TEM, samples were similarly fixed in 2.5% glutaraldehyde and postfixed in 1% osmium tetroxide, followed by dehydration in a graded ethanol series. After infiltration and embedding in epoxy resin, ultrathin sections (approximately 70 nm) were prepared using an ultramicrotome (Ultracut S, Leica, Austria) and stained with uranyl acetate and lead nitrate. TEM observations were performed on a JEM-1200EX transmission electron microscope (JEOL, Tokyo, Japan) at an accelerating voltage of 80 kV. These procedures allowed detailed visualization of fungal hyphae, extracellular matrix, and other ultrastructural features of the fungus balls.

### Galactomannan assay

Fungus balls collected on weeks 0 to 16 post-implantation were homogenized in 1 mL of saline with a 6 mm stainless steel bead and 200 mg of 0.6 mm zirconia/silica beads, using a BMS-M10N21 homogenizer at 1,500 rpm for 5 min. After centrifugation, the supernatant was collected. The relative fungal content for each fungus ball was determined by measuring GM content in the supernatant (36), using the Platelia *Aspergillus* enzyme immunoassay kit (Bio-Rad) according to the manufacturer’s instructions. GM was quantified for samples collected up to week 16; later time points were not assayed**.**

### Vascular endothelial growth factor analysis

Supernatants from the homogenized fungus balls were also analyzed for VEGF content. VEGF levels were assessed using a custom Mouse Cytokine/Chemokine Panel (Merck Millipore, USA) according to the manufacturer’s instructions. Fluorescence was measured with a Luminex 200 instrument (Luminex Corporation, USA), and data were analyzed using MILLIPLEX Analyst (version 5.1). VEGF levels were normalized to the GM index for each sample.

### *In vitro* lipid droplet staining

RAW264.7 cells (mouse peritoneal macrophage cell line) were maintained in Dulbecco’s Modified Eagle’s Medium with 10% fetal bovine serum at 37°C in 5% CO_2_. Cells (1.5 × 10^5^/well) were seeded in 24-well plates for 20 h and then incubated for 24 h with 1.2 × 10^6^ heat-killed *A. fumigatus* hyphae at a multiplicity of infection (MOI) of 8. Controls received medium alone. Lipid droplet formation was visualized by Oil Red O staining according to the manufacturer’s protocol (Bio Mirai Koubou, Tokyo, Japan). Ten random ×400 fields per well were imaged, and lipid droplet area was quantified using Fiji (ImageJ v2.9).

### *In vitro* cytotoxic analysis of *A. fumigatus* dead hyphae on RAW264.7 cells

The extent of host cell damage induced by killed *A. fumigatus* hyphae was determined by a ^51^Cr release assay and an XTT assay as previously described (37, 38). Two laboratory strains, Af293 and CEA10, were used. Conidia were incubated in potato dextrose broth at 37°C for 8 h to generate hyphae, which were then killed by heating at 95°C for 10 min. Heat-killed hyphae were rinsed in PBS before use.

RAW264.7 cells (1.25 × 10^5^/well) were labeled overnight with ^51^Cr in a 48-well plate. The next day, cells were washed in PBS to remove unincorporated ^51^Cr, and 3.75 × 10^6^ heat-killed hyphae (MOI = 30) were added in 500 µL of medium per well. After 24 h of incubation, 250 µL of supernatant was collected. The cells were lysed with 6 N NaOH and washed with RadiacWash (Biodex Medical Systems). Gamma radiation in both fractions (supernatant and lysed cells) was counted. Spontaneous ^51^Cr release was measured from uninfected RAW264.7 cells. The percent specific release (i.e., cell damage) was calculated with a previously described formula (39).

RAW264.7 cells (6.3 × 10^4^/well) were seeded in a 96-well plate the night before the assay. The following day, cells were washed in PBS, and 1.9 × 10^6^ heat-killed hyphae (MOI = 30) were added in 100 µL of medium per well. After 24 h, the XTT solution (American Type Culture Collection, #30-1011K) was added. The plate was incubated for 60 min, and 120 µL of the supernatant was transferred to a fresh plate for spectrophotometric measurement (OD_475nm − OD_660nm). The percent damage was calculated relative to control cells not exposed to hyphae: Percent damage = 100 × (1 – [sample OD/control OD]). Unless stated otherwise, all *in vitro* assays were performed in technical triplicate and reproduced in at least three independent biological experiments.

### Statistical analyses

Statistical significance was assessed with the Mann-Whitney U test for two-group comparisons and with the Kruskal-Wallis test, followed by Dunn’s multiple-comparison test for analyses involving three or more groups. A *P* value <0.05 was considered statistically significant. All analyses were performed using JMP Pro 17 (ver. 17.1; SAS Institute, Cary, NC, USA).

## Data Availability

All data sets supporting the conclusions of this article are included in the main text or the Supplemental figures. Full-resolution histology and electron microscopy images and the ImageJ/Fiji analysis macro are available from the corresponding author upon reasonable request.

## SUPPLEMENTAL MATERIAL

Supplemental figures spectrum.03467-25-s0001.pdf Fig. S1 and S2.

ASM does not own the copyrights to Supplemental Material that may be linked to, or accessed through, an article. The authors have granted ASM a non-exclusive, world-wide license to publish the Supplemental Material files. Please contact the corresponding author directly for reuse.
