# Supplementary material for: Non-viable Aspergillus fumigatus promotes chronic inflammation and angiogenesis in a murine fungus ball model
Source: Microbiol Spectr. 2026 Mar 24;14(5):e03467-25. doi: 10.1128/spectrum.03467-25 (PMC13141880; doi:10.1128/spectrum.03467-25)
Supplement: Supplemental figures — Fig. S1 and S2. [file spectrum.03467-25-s0001.pdf]

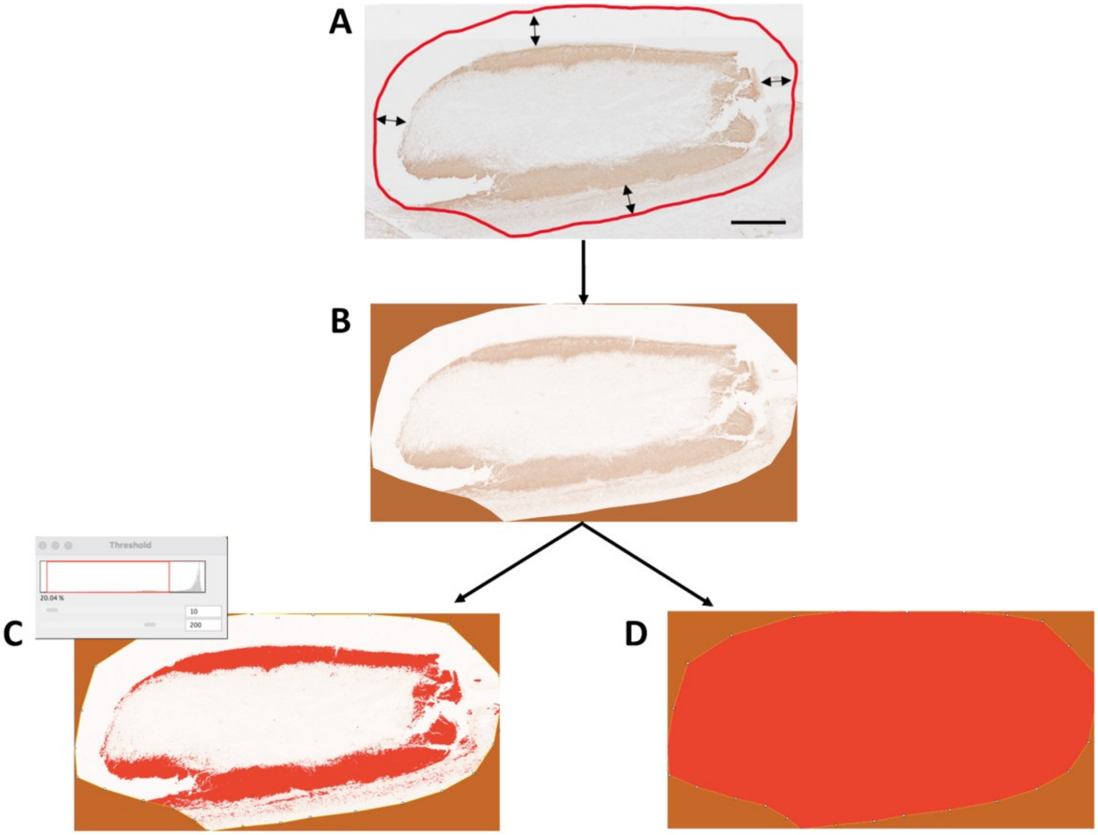

FIG S1 ImageJ analysis and quantification of positive-staining area. (A) Whole-slide scan (GMS/IHC, Oil Red O, or Sirius Red) showing a fungus ball (outlined in red). A freehand selection was placed 500 µm outside the fungal mass in all directions (black double-headed arrows) to define the analysis region. (B) Area outside the selection was removed using the Clear Outside command, leaving only the fungus ball plus a 500-µm margin on a uniform background. (C) Positive staining (DAB, Oil Red O, or Sirius Red) was isolated with the Color Threshold tool in Fiji/ImageJ; pixels above the threshold are pseudocolored red. (D) The same selection was converted to a binary mask to obtain the total area (fungus ball + margin). The percentage positive area was calculated as (positive-stained area / total selected area) × 100.

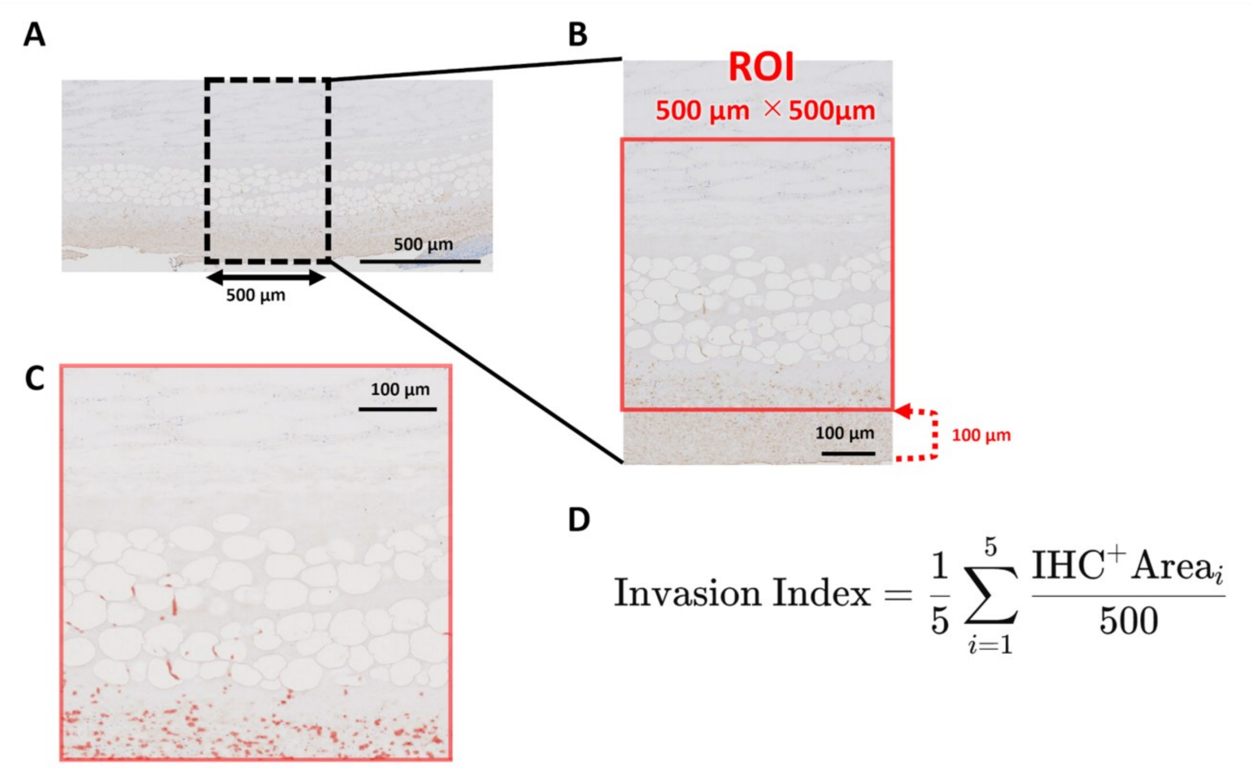

FIG S2 Quantitative image-analysis workflow for assessing *Aspergillus* tissue invasion. (A) Whole-slide IHC image showing a continuous 500-µm fungus–tissue interface (black dashed box). (B) Enlarged view of the boxed area. A virtual boundary 100 µm inside the tissue (red dashed line) was generated, and one of the five 500 µm × 500 µm regions of interest (ROIs; red box) is illustrated. (C) Example ROI after color thresholding; IHC-positive pixels are pseudocolored red. (D) Invasion Index formula, where IHC+ area_i is the positive area in the i-th ROI.
